# Microbiologically confirmed infections and antibiotic-resistance in a national surveillance study of hospitalised patients who died with COVID-19, Italy 2020–2021

**DOI:** 10.1186/s13756-022-01113-y

**Published:** 2022-05-21

**Authors:** Marco Floridia, Marina Giuliano, Monica Monaco, Luigi Palmieri, Cinzia Lo Noce, Anna Teresa Palamara, Annalisa Pantosti, Silvio Brusaferro, Graziano Onder, The Italian National Institute of Health COVID-19 Mortality Group, Luigi Palmieri, Elvira Agazio, Pierfrancesco Barbariol, Antonino Bella, Eva Benelli, Luigi Bertinato, Matilde Bocci, Stefano Boros, Marco Bressi, Giovanni Calcagnini, Marco Canevelli, Federica Censi, Alessandra Ciervo, Elisa Colaizzo, Roberto Da Cas, Martina Del Manso, Corrado Di Benedetto, Chiara Donfrancesco, Massimo Fabiani, Francesco Facchiano, Marco Floridia, Fabio Galati, Marina Giuliano, Tiziana Grisetti, Cecilia Guastadisegni, Ilaria Lega, Cinzia Lo Noce, Pietro Maiozzi, Valerio Manno, Margherita Martini, Marco Massari, Alberto Mateo Urdiales, Eugenio Mattei, Claudia Meduri, Paola Meli, Francesca Menniti Ippolito, Giada Minelli, Graziano Onder, Daniele Petrone, Patrizio Pezzotti, Flavia Pricci, Ornella Punzo, Federica Quarata, Valeria Raparelli, Flavia Riccardo, Simone Rocchetto, Chiara Sacco, Paolo Salerno, Giulia Sarti, Debora Serra, Stefania Spila Alegiani, Matteo Spuri, Marco Tallon, Manuela Tamburo De Bella, Dorina Tiple, Marco Toccaceli Blasi, Federica Trentin, Brigid Unim, Luana Vaianella, Nicola Vanacore, Maria Fenicia Vescio, Emanuele Rocco Villani, Liliana Elena Weimer, Silvio Brusaferro

**Affiliations:** 1 National Center for Global Health, Istituto Superiore Di Sanità, Viale Regina Elena 299, 00161 Rome, Italy; 2 Department of Infectious Diseases, Istituto Superiore Di Sanità, Rome, Italy; 3 Department of Cardiovascular, Endocrine-Metabolic Diseases and Aging, Istituto Superiore Di Sanità, Rome, Italy; 4 Istituto Superiore Di Sanità, Rome, Italy

**Keywords:** COVID-19, Co-infections, Secondary infections, Bloodstream infections, Lower respiratory tract infections, Bacterial infections, Fungal infections, Antimicrobial resistance

## Abstract

**Background:**

Patients hospitalised for COVID-19 may present with or acquire bacterial or fungal infections that can affect the course of the disease. The aim of this study was to describe the microbiological characteristics of laboratory-confirmed infections in hospitalised patients with severe COVID-19.

**Methods:**

We reviewed the hospital charts of a sample of patients deceased with COVID-19 from the Italian National COVID-19 Surveillance, who had laboratory-confirmed bacterial or fungal bloodstream infections (BSI) or lower respiratory tract infections (LRTI), evaluating the pathogens responsible for the infections and their antimicrobial susceptibility.

**Results:**

Among 157 patients with infections hospitalised from February 2020 to April 2021, 28 (17.8%) had co-infections (≤ 48 h from admission) and 138 (87.9%) had secondary infections (> 48 h). Most infections were bacterial; LRTI were more frequent than BSI. The most common co-infection was pneumococcal LRTI. In secondary infections, Enterococci were the most frequently recovered pathogens in BSI (21.7% of patients), followed by *Enterobacterales*, mainly *K. pneumoniae*, while LRTI were mostly associated with Gram-negative bacteria, firstly *Enterobacterales* (27.4% of patients, *K. pneumoniae* 15.3%), followed by *A. baumannii* (19.1%). Fungal infections, both BSI and LRTI, were mostly due to *C. albicans*. Antibiotic resistance rates were extremely high in Gram-negative bacteria, with almost all *A. baumannii* isolates resistant to carbapenems (95.5%), and *K. pneumoniae* and *P. aeruginosa* showing carbapenem resistance rates of 59.5% and 34.6%, respectively.

**Conclusions:**

In hospitalised patients with severe COVID-19, secondary infections are considerably more common than co-infections, and are mostly due to Gram-negative bacterial pathogens showing a very high rate of antibiotic resistance.

**Supplementary Information:**

The online version contains supplementary material available at 10.1186/s13756-022-01113-y.

## Introduction

SARS-CoV-2 infection is in most of the cases asymptomatic or characterized by mild to moderate clinical symptoms that do not require hospital admission. In a significant proportion of cases, however, the infection may progress to severe disease, mostly because of pulmonary involvement, requiring hospitalisation in COVID-19 dedicated medical units or, particularly when invasive respiratory support is needed, in intensive care units (ICU) [1].

In this setting, hospitalisation may be prolonged for several weeks, facilitating the occurrence of infections that may increase the severity of the disease and the risk of a fatal outcome, especially in individuals with advanced age, frailty, or severe comorbidity [2]. The Italian National COVID-19 mortality surveillance has reported a 19% prevalence of infections among patients who died in hospital with confirmed SARS-CoV-2 [3]. This rate, although based on deceased patients only, is consistent with recent data that showed a prevalence from 3.0 to 3.7% for co-infections [4–7] and of 14.5% for secondary infections in hospitalised patients with COVID-19 during the first wave of the pandemic [4].

Antimicrobial resistance (AMR) in hospitalised patients represents another challenge for the health systems worldwide. Italy is one of the European countries with the highest prevalence of infections due to antibiotic-resistant bacteria, that often complicate the course of admissions for other conditions [8]. Effective treatment of such infections, due to the scarcity or complete lack of active antibiotics, is problematic, and infections caused by multi-drug resistant organisms (MDRO), especially Gram-negative pathogens, are burdened with high mortality [9, 10].

AMR and COVID-19 have intermingled in the last months. The important changes introduced during the COVID-19 pandemic in preventive measures, hospital organization and patient care, including widespread use of antibiotics and corticosteroids, may have influenced the rates and characteristics of AMR [11, 12].

Defining the aetiology of bacterial and fungal co-infections and secondary infections in hospitalised patients with COVID-19 and the resistance profile of the microorganisms involved may therefore be relevant to define treatment strategies aimed at preventing or reducing morbidity and mortality in patients with SARS-CoV-2 infection. The present study was conducted with the aim to characterize the blood and lower respiratory tract infections in patients deceased with COVID-19 and the resistance profile of the microorganisms detected.

## Methods

The present study was nested in the Italian National COVID-19 Surveillance on causes of death in individuals with PCR-confirmed SARS-CoV-2 infection. Within this surveillance, coordinated by the Istituto Superiore di Sanità (ISS, the Italian National Institute of Health), all Italian Regions and Autonomous Provinces send to the ISS the hospitalisation records of patients with PCR-confirmed SARS-CoV-2 infection deceased in hospital. A random sample of records, representative of the regional distribution of COVID-19 related deaths, is reviewed at ISS by a team of medical doctors, who enter selected data in the surveillance database. This database includes information on several items, including comorbidities, admission to Intensive Care Unit (ICU), time interval from hospitalisation to death, occurrence of complications, and cause of death as reported on the official death certificate [13]. For the present study, hospital charts documenting infections diagnosed during hospital stay were further reviewed by two of the authors, who extracted additional information on early treatment with antibiotics and steroids, time and type of clinical samples collected for microbiological investigations, microorganisms detected, resistance profiles, time from hospitalisation and from ICU admission to development of co-infections and secondary infections.

Eligibility criteria for this study were represented by presence of laboratory-confirmed bloodstream or lower respiratory tract infections (BSI and LRTI, respectively), defined as positive cultures for bacteria or fungi of blood, bronchial/endotracheal aspirate or broncoalveolar lavage, or positive urinary antigen test for *Streptococcus pneumoniae* or *Legionella* *pneumophila*. For lower respiratory tract samples, cultures yielding a microbial count of less than 10^4^ CFU/ml were excluded. Common skin contaminants, such as coagulase-negative S*taphylococcus spp., Corynebacterium spp.* and *Bacillus spp*. were not considered. Infections diagnosed only on a clinical basis (without microbiological confirmation) or occurring in other sites, such as the urinary tract, skin or other tissues, were also excluded.

Infections were categorized by microorganism detected, diagnostic procedure (blood culture, lower respiratory tract culture, urinary antigen test), ward of occurrence (intensive care or other clinical departments), and time of occurrence (considering co-infections those diagnosed from samples collected within 48 h from hospital admission, and secondary infections those diagnosed from samples collected thereafter).

For antibiotic resistance, we analysed the MDRO considered as critical or high priority by WHO, with the corresponding most relevant antibiotic resistance traits [14]. More specifically, the MDRO analysed were: carbapenem-resistant *Acinetobacter baumannii* (CRAB); carbapenem-resistant *Pseudomonas aeruginosa*; carbapenem-resistant and/or 3^rd^ generation cephalosporin resistant *Enterobacterales* (CRE and/or 3GCRE, respectively), vancomycin-resistant *Enterococcus faecium* or *Enterococcus faecalis* (VRE), and methicillin-resistant *Staphylococcus aureus* (MRSA).

The study included COVID-19 patients admitted to hospital from February 2020 to April 2021; at the time of analysis this period of observation was divided in two phases (February 2020-September 2020 and October 2020-April 2021), roughly corresponding to the first and second wave of the COVID-19 pandemic in Italy [3].

The main intent of the study was descriptive. We defined a priori an arbitrary sample size of 150 eligible cases, considering this size adequate for descriptive purposes, and stopped data extraction from the series analysed when this number was reached. Quantitative variables were compared using the Mann–Whitney U-test or Student T test and categorical variables with the chi-square test or the Fisher test, as appropriate. Odds ratios (OR) with 95% confidence intervals (CI) were calculated. *P* values < 0.05 were considered statistically significant. All analyses were performed using the SPSS software, version 27.0 (IBM Corp, 2017, Armonk, NY, USA).

The collection and scientific dissemination of data related to COVID-19 epidemics by the ISS and other public health bodies was authorized on February 27th, 2020, by the Italian Presidency of the Council of Ministers [15].

## Results

We analysed 157 eligible patients with laboratory-confirmed infections who were hospitalised between February 2020 and April 2021 and deceased between March 2020 and May 2021. These cases represent 11.3% of the 1390 clinical records with reported superinfections included in the ISS mortality database as of April 28, 2021 (shown in Additional file 1: Table S1). The general characteristics of the study population are reported in Table 1.

**Table 1:** Population characteristics

Number of patients evaluated	157	
Age (median, interquartile range)	71 (63–79)	
	N	%
*Sex*		
Male	117	74.5
Female	40	25.5
*Patient location before admission*		
Home	123	78.3
Other hospital	18	11.5
Long-term care facility	11	7.0
Other	5	3.2
*Geographical area (Italy)*		
North	111	70.7
Center	27	17.2
South	19	12.1
*Period of hospitalisation*		
February 2020-September 2020	72	45.9
October 2020-April 2021	85	54.1
*Comorbidities*		
Chronic respiratory disease	32	20.4
Neoplastic disease	19	12.1
Diabetes	42	26.8
Chronic renal failure	26	16.6
*Antibiotics in the first 48 h from admission*		
Any	95	60.5
Azithromycin	40	25.5
Ceftriaxone	43	27.4
Piperacillin-tazobactam	22	14.0
Others	26	16.6
Steroids in the first 48 h from admission	72	45.9
Admission to Intensive Care Unit (ICU)	111	70.7
*Level of care at first positive blood culture*		
ICU	51	60.7
Other wards	33	39.3
*Level of care at first positive lower respiratory tract culture*		
ICU	95	88.8
Other wards	12	11.2
*Level of care at first positive urinary antigen test (for S. pnemoniae or L. pneumophila)*		
ICU	3	18.8
Other wards	13	81.2
Sepsis reported as a cause of death in the death certificate	57	36.3

Median age of the population studied was 71 years; 74.5% of cases were males, 78.3% were living at their homes before hospital admission, and 70.7% lived in North Italy. As for co-morbidities, one quarter of the patients had diabetes, 20% chronic respiratory disease, 16% chronic renal failure, and 12% neoplastic disease. In the first 48 h from admission, 60% received antibiotics (most commonly azithromycin or ceftriaxone) and 45% steroids. In 57 patients (36.3%), sepsis was reported as a contributory cause of death in the death certificate. The cases analysed were evenly distributed in the two periods considered (Table 1). No significant differences were found between the two periods in patient age (*p* = 0.331, T test), sex (*p* = 0.621, chi-square test), patient location before hospital admission (*p* = 0.818, chi-square test), geographical area (*p* = 0.461, chi-square test) or antibiotic treatment in the first 48 h from admission (*p* = 0.853, chi-square test).

Most of the patients (70.7%) were admitted to ICU during the hospital stay. The majority of the infections detected by culture were diagnosed in ICU (60.7% of BSI and 88.7% of LRTI).

**Fig. 1:**
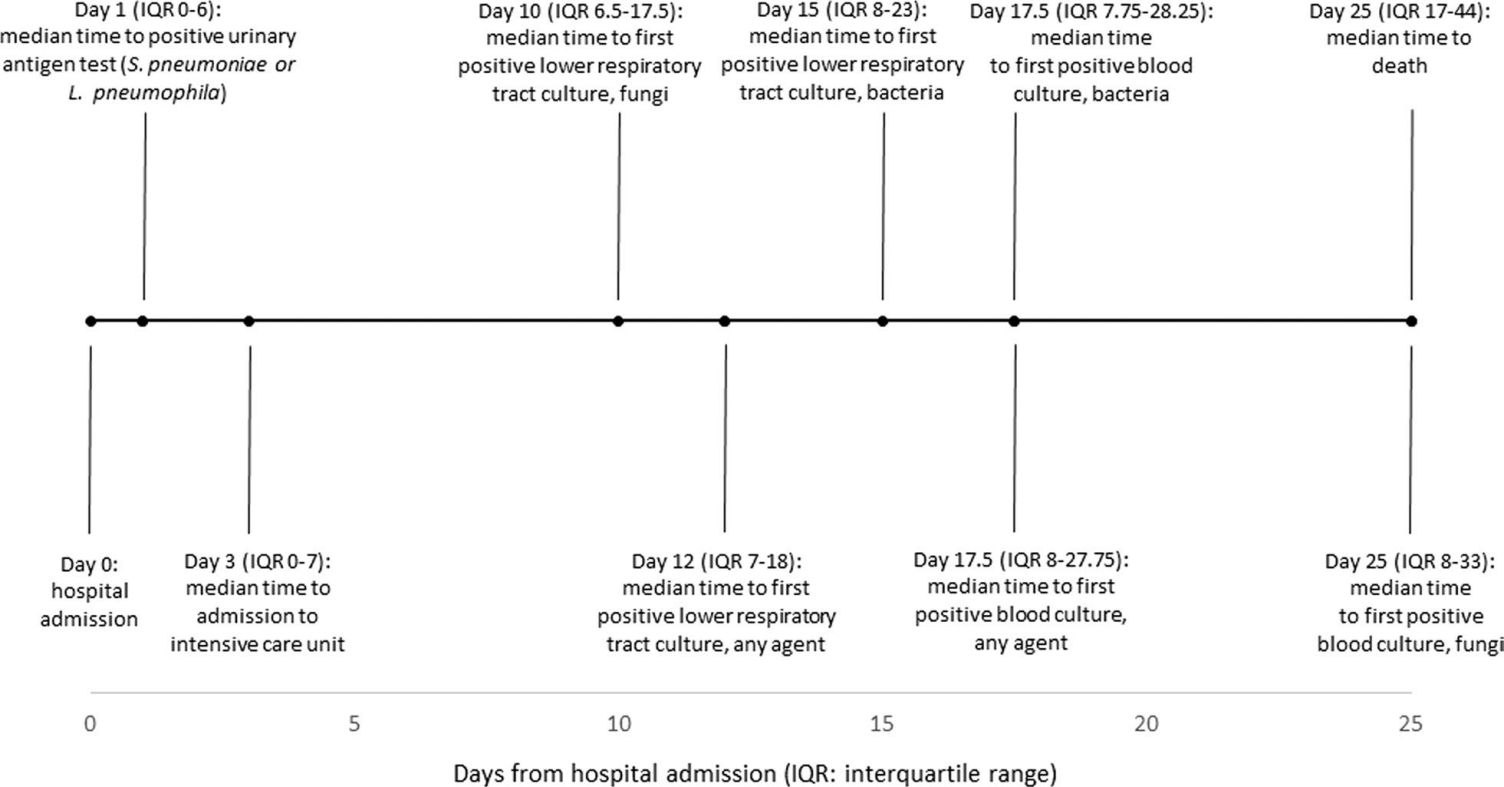
Median time to events from hospital admission

Time of occurrence of infections with respect to hospital admission is reported in Fig. 1. Early events were detection of *S. pneumoniae* or *L. pneumophila* infections by urinary antigen test (median interval from admission to either *S. pneumoniae* or *L. pneumophila* antigen detection 1 day, IQR 0–6) and admission to ICU (3 days, IQR 0–7). Median time from hospital admission to first positive lower respiratory culture, first positive blood culture and death were 12 days (IQR 7–18), 17.5 days (IQR 8–27.75) and 25 days (IQR 17–44), respectively.

One-hundred-seven patients (68.1%) had at least one positive culture from a deep respiratory tract sample yielding a relevant bacterial and/or fungal pathogen, 84 patients (53.5%) had at least one positive significant blood culture, and 16 (10.2%) a positive urinary antigen test for either *S. pneumoniae* (n = 15) or *L. pneumophila* (n = 1). Overall, 137 patients had one or more bacterial infections, with a cumulative number of 245 bacterial isolates detected, and 61 patients had fungal infections, with 64 isolates detected.

The general characteristics of infections by microorganism detected site of infection (BSI/LRTI), and time of occurrence (co-infections or secondary infections), are reported in Table 2. Only few patients (28/157, 17.8%) had co-infection detected at hospital admission or within 48 h. Most of these co-infections (24/28) were bacterial, represented by pneumococcal pneumonia, diagnosed by the urinary antigen test, and BSI, mainly due to *S. aureus* (Table 2). Fungal infections in the first 48 h from admission were uncommon, being detected in four patients only.

**Table 2:** Patients with microbiologically confirmed infections by microorganism, time from hospital admission and site of infection

Microorganism detected	Patients (n, %) with infection at any time from hospital admission^			Patients (n, %) with infection within 48 h from admission (co-infection)			Patients (n, %) with infection after 48 h from admission (secondary infection)		
	BSI, LRTI, or both	BSI	LRTI	BSI, LRTI, or both	BSI	LRTI	BSI, LRTI, or both	BSI	LRTI
*Acinetobacter baumannii*	37 (23.5)	16 (10.2)	30 (19.1)	2 (1.3)	2 (1.3)	0	35 (22.3)	14 (8.9)	30 (19.1)
*Enterobacterales*	58 (36.9)	23 (14.6)	43 (27.4)	4 (2.5)	4 (2.5)	0	54 (34.4)	19 (12.1)	43 (27.4)
*Escherichia coli*	12 (7.6)	6 (3.8)	6 (3.8)	3 (1.9)	3 (1.9)	0	9 (5.7)	3 (1.9)	6 (3.8)
*Klebsiella pneumoniae*	30 (19.1)	13 (8.3)	24 (15.3)	1 (0.6)	1 (0.6)	0	29 (18.5)	12 (7.6)	24 (15.3)
Other *Enterobacterales*°	23 (14.6)	4 (2.5)	19 (12.1)	0	0	0	23 (14.6)	4 (2.5)	19 (12.1)
*Enterococcus* spp.	43 (27.4)	36 (22.9)	9 (5.7)	2 (1.3)	2 (1.3)	0	41 (26.1)	34 (21.7)	9 (5.7)
*Enterococcus faecalis*	27 (17.2)	22 (14.0)	6 (3.8)	2 (1.3)	2 (1.3)	0	25 (15.9)	20 (12.7)	6 (3.8)
*Enterococcus faecium*	16 (10.2)	14 (8.9)	3 (1.9)	0	0	0	16 (10.2)	14 (8.9)	3 (1.9)
Legionella pneumophila*	1 (0.6)	0	1 (0.6)	0	0	0	1 (0.6)	0	1 (0.6)
*Pseudomonas aeruginosa*	24 (15.3)	8 (5.1)	21 (13.4)	0	0	0	24 (15.3)	8 (5.1)	21 (13.4)
*Staphylococcus aureus*	33 (21.0)	13 (8.3)	21 (13.4)	7 (4.5)	3 (1.9)	4 (2.5)	26 (16.6)	10 (6.4)	17 (10.8)
Streptococcus pneumoniae**	16 (10.2)	1 (0.6)	15 (9.6)	10 (6.4)	0	10 (6.4)	6 (3.8)	1 (0.6)	5 (3.2)
Other bacteria^@^	16 (10.2)	4 (2.5)	12 (7.6)	0	0	0	16 (10.2)	4 (2.5)	12 (7.6)
*Candida* spp.	50 (31.8)	17 (10.8)	35 (22.3)	1 (0.6)	1 (0.6)	0	49 (31.2)	16 (10.2)	35 (22.3)
*Candida albicans*	36 (22.9)	9 (5.7)	28 (17.8)	0	0	0	36 (22.9)	9 (5.7)	28 (17.8)
Other *Candida* spp.^†^	17 (10.8)	7 (4.5)	10 (6.4)	1 (0.6)	1 (0.6)	0	16 (10.2)	6 (3.8)	10 (6.4)
Other fungi^#^	13 (8.3)	1 (0.6)	12 (7.6)	3 (1.9)	1 (0.6)	2 (1.3)	10 (6.4)	0	10 (6.4)

*BSI* bloodstream infection, *LRTI* lower respiratory tract infection: infection detected by culture of bronchial/endotracheal aspirate or of bronchoalveolar lavage (107 patients) or by urinary antigen test (16 patients)

^9 patients had both co-infection and secondary infection

°Other *Enterobacterales* (all secondary infections): 9 *Klebsiella aerogenes* (3 BSI, 6 LRTI); 1 *Klebsiella ornithinolytica* (bloodstream); 2 *Klebsiella oxytoca* (LRTI); 6 *Serratia marcescens* (LRTI); 3 *Enterobacter cloacae* (LRTI), 1 *Providencia stuartii* (LRTI); 1 *Proteus mirabilis* (LRTI)

^*^Detected by urinary antigen test

^**^15 Detected by urinary antigen test, 1 by blood culture

^@^Other bacteria (all secondary infections): 7 *Stenotrophomonas maltophilia* (LRTI); 1 *Morganella morganii* (LRTI); 2 *Bacteroides fragilis* (bloodstream); 1 *Sphingomonas mucosissima* (BSI); 1 *Chrysobacterium meningiosepticum* (LRTI); 1 *Haemophilus influenzae* (LRTI; 2 *Burkholderia gladioli* (1 BSI, 1 LRTI); 1 *Branhamella catarrhalis* (LRTI)

^†^Other *Candida* spp.: 7 *Candida glabrata* (2 bloodstream, 1 co-infection and 1 secondary infection; 5 LRTI, all secondary infections); 7 *Candida tropicalis* (all secondary infections, 2 bloodstream, 5 LRTI); 3 *Candida parapsilosis* (all bloodstream secondary infections)

^#^Other fungi: 10 *Aspergillus* spp. (2 co-infections, LRTI, 8 secondary infections, LRTI; 1 *Cryptococcus* (co-infection, bloodstream); 1 *Pneumocystis* (secondary infection, LRTI); 1 unspecified (secondary infection, LRTI)

Secondary infections represented the largest burden of infections, involving 138 of 157 patients (87.9%). Most of secondary infections were LRTI, diagnosed by culture (in 103/138 patients, 74.6%) or, much less commonly, by urinary antigen test. BSI as secondary infections were detected in half of the patients (73/138 patients, 52.9%). Nine patients (5.7%) were diagnosed with both co-infection and secondary infection.

The bacterial pathogens recovered from secondary infections showed a different distribution between BSI and LRTI. In BSI, Enterococci were the most frequently recovered pathogens (in 21.7% of patients: *E. faecalis* 12.7%, *E. faecium* 8.9%), followed by *Enterobacterales* (12.1%, mainly *K. pneumoniae*, 7.6%), *A. baumannii* (8.9%), *S. aureus* (6.4%) and *P. aeruginosa* (5.1%). In LRTI there was a clear predominance of Gram-negative pathogens, with the most common species represented by *A. baumannii* (in 19.1% of patients)*,* followed by *K. pneumoniae* (15.3%) and *P. aeruginosa* (13.4%); *S. aureus* was found in 10.8% of patients. Fungal infections were mostly due to *C. albicans*, for both BSI (10.2% of patients) and LRTI (22.3% of patients).

Forty-five patients (28.7%) had positive cultures in both blood and lower respiratory samples, and 25 patients had the same microorganism (8 patients had *A. baumannii*, 5 had *P. aeruginosa*, 4 had *K. pneumoniae*, and 8 other species). Multiple pathogens were concurrently or sequentially detected in 31/84 (36.9%) patients with blood infections and in 61/107 (57.0%) patients with positive lower respiratory tract cultures.

We found no differences in lag time from hospitalisation to secondary infections by geographical area (North Italy versus Centre/South, *p* = 0.788, Mann–Whitney U test), time period (first versus second pandemic wave, *p* = 0.650, Mann–Whitney U test) or microbial agent involved (bacterial versus fungal, *p* = 0.307, Mann–Whitney U test). We also found no significant difference in risk of primary or secondary infections according to comorbidities such as chronic respiratory infections, neoplastic disease, diabetes or chronic renal failure (data not shown).

Antimicrobial resistance was evaluated for bacterial pathogens only, because susceptibility tests to antifungal agents for *Candida* isolates were infrequently performed. Susceptibility tests were available for 95.1% (97/102) of bacterial blood isolates and 93.0% (133/143) of bacterial respiratory isolates, corresponding to a total of 230 isolates from 121 patients. Seventy-five patients (62.0%) had at least one resistant bacterial isolate, with a significant higher rate in patients hospitalised in Central/South Italy compared to North Italy (33/41, 80.5% vs. 42/80, 52.5%, *p* = 0.002) and a non-significant trend for a higher rate in patients hospitalised during the second wave compared to the first wave (44/65, 67.7% vs. 31/56, 55.3%, *p* = 0.16). The most relevant antibiotic resistance traits of the 230 bacterial isolates are summarized in Table 3. Carbapenem resistance (CR) was nearly total in *A. baumannii* (95.5% of isolates), very high in *K. pneumoniae* (59.5%), and common in *P. aeruginosa* (34.6%). Resistance to 3^rd^-generation cephalosporins was highly prevalent in *K. pneumoniae* (86.5%) and *E. coli* (70.0%). Half of *S. aureus* isolates were methicillin-resistant, whereas for *Enterococcus spp*. the most relevant antibiotic resistance trait (vancomycin resistance) was not detected. Occurrence of resistance to 3^rd^ generation cephalosporins in *Enterobacterales* was not associated with use of ceftriaxone (odds ratio for resistance: 1.773, 95% CI 0.396–7.932, *p* = 0.454) or of corticosteroids (odds ratio for resistance: 1.630, 95% CI 0.411–6.459, *p* = 0.487) in the first 48 h from admission.

**Table 3:** Bacterial species and resistance traits of 230 isolates obtained from blood and lower respiratory tract of 121 patients

	All infections		Blood		Lower respiratory tract	
	N of isolates	Resistant, n (%)	N of isolates	Resistant n (%)	N of isolates	Resistant n (%)
*Enterobacterales* (all)	67	46 (68.7) 3GCR^a^	22	15 (68.2) 3GCR	45	31 (68.9) 3GCR
		23 (34.3) CRE^b^		8 (36.4) CRE		15 (33.3) CRE
* Klebsiella pneumoniae*	37	32 (86.5) 3GCR	12	10 (83.3) 3GCR	25	22 (88.0) 3GCR
		22 (59.5) CR^c^		8 (66.6) CR		14 (56.0) CRE
* Escherichia coli*	10	7 (70.0) 3GCR	6	3 (50.0) 3GCR	4	4 (100) 3GCR
		0 (0) CR		0 (0) CR		0 (0) CRE
Other *Enterobacterales*	20	7 (35.0) 3GCR	4	2 (50.0) 3GCR	16	5 (31.2) 3GCR
		1 (5.0) CR		0 (0) CR		1 (6.2%) CR
*Acinetobacter baumannii*	45	43 (95.5) CRAB^d^	16	15 (93.7) CRAB	29	28 (93.3) CRAB
*Pseudomonas aeruginosa*	26	9 (34.6) CR	7	1 (12.5) CR	19	8 (42.1) CR^e^
*Enterococci* (all)	43	0 (0) VRE^f^	34	0 (0) VRE	9	0 (0) VRE
* Enterococcus faecalis*	28	0 (0) VRE	22	0 (0) VR	6	0 (0) VRE
* Enterococcus faecium*	15	0 (0) VRE	12	0 (0) VRE	3	0 (0) VRE
*Staphylococcus aureus*	34	17 (50.0) MRSA^g^	13	7 (53.8) MRSA	21	10 (47.6) MRSA
Other bacterial species	15	4 (26.6) MDRO^h^	5	1 (20.0) MDRO^i^	10	3 (30.0) MDRO^j^

^a^3GCR: resistant to 3rd generation cephalosporins

^b^CRE: *Enterobacterales* resistant to carbapenems

^c^CR: resistant to carbapenems

^d^CRAB: *Acinetobacter baumannii* resistant to carbapenems

^e^Including 2 cases with carbapenem resistance acquired during carbapenem treatment

^f^VRE: *Enterococcus* spp. resistant to vancomycin

^g^MRSA: methicillin/oxacillin resistant *Staphylococcus aureus*

^h^MDRO: resistant to ceftazidime and carbapenems

^i^1 *Bacteroides fragilis* resistant to ceftazidime and carbapenems

^j^2 *Stenotrophomonas maltophilia* and 1 *Crysobacterium meningosepticum* resistant to ceftazidime and carbapenems

## Discussion

This descriptive study reports the characteristics of 157 patients with COVID-19 deceased in hospitals with microbiologically confirmed infections (co-infections or secondary infections), the microorganisms associated with these infections and the antibiotic resistance traits of the bacterial pathogens. Analysis was limited to BSI and LRTI, considered to be more likely related to COVID-19 than other types of infections [16]. Although this study included only deceased patients, the picture obtained is in line with a recent meta-analysis and other studies [4, 6, 16], and indicates that, among microbiologically confirmed infections, co-infections are a minority and are mostly due to *S. pneumoniae* and *S. aureus*, pathogens known to be responsible for co-infections also in pandemic and seasonal influenza [17]. In a multicentre study from United Kingdom, co-infections represented 30% of all infections; the most common microorganisms obtained by culture were *S. aureus* in LRTI and *E. coli* in BSI, but the results of urinary antigen tests were excluded from the analysis [16]. Urinary antigen tests played a major role in the diagnostics of S*. pneumoniae* infection in our study, with 15/16 cases diagnosed using urinary antigen tests and only one by blood culture. Urinary antigen test was usually performed soon after hospital admission, leading to an earlier diagnosis of pneumococcal infection compared to other infections. Urinary antigen test for S*. pneumoniae* is considered to be sensitive and highly specific in the adult population [18], and is usually performed without further microbiological workout. Low rates of detection of *S. pneumoniae* in culture in this study may be explained by the difficulty of isolating *S. pneumoniae* in culture in patients with previous exposure to antibiotics and by preferential use of urinary antigen test for diagnosis.

Secondary infections represent the major burden of infections and are more common in patients in ICU. In our study, 70% of the patients were admitted to ICU and likely received mechanical ventilation. The microorganisms detected in secondary infections were pathogens frequently responsible for healthcare-associated infections, with the associated antibiotic resistance traits. In LRTI, antibiotic-resistant, Gram-negative pathogens predominated: *Enterobacterales*, primarily *K. pneumoniae,* followed by *A. baumannii*, and *P. aeruginosa*. BSI were diagnosed later than LRTI during hospitalization, and the most commonly isolated microorganisms were enterococci (both *E. faecalis* and *E. faecium*), followed by *A. baumannii* and *K. pneumoniae*. *S. aureus* was detected in a non-negligible percentage from both sample types. The distribution of the pathogens detected was similar to that described in other studies [2, 16], with two noteworthy differences: *E. coli* was rarely isolated, probably because urinary tract infections were not taken into account, and *A. baumannii* was exceedingly frequent. *A. baumannii* was a frequent and increasing cause of infections in critically ill patients in Italy prior to the COVID-19 pandemic [19], and its role in infections and mortality in COVID-19 patients has been shown [20]. The high prevalence of BSI due to enterococci, observed also in other studies [16], could be ascribed to the translocation of gut bacteria secondary to intestinal mucosa damage due to SARS-CoV-2 [21].

Fungal pathogens, commonly *C. albicans,* seem to play a minor role, although they were detected especially in secondary LRTI. In particular, in almost one quarter of the patients *Candida* spp. was recovered from low respiratory tract cultures. Although we did not consider sputum samples but only cultures of bronchial/endotracheal aspirate or of bronchoalveolar lavage, and even if we excluded cultures yielding a microbial count of less than 10^4^ CFU/ml, we cannot exclude that positive LRT cultures for *Candida* represent colonization and not infection [22]. However, given the predominant role of pulmonary disease in determining severe COVID-19 disease and mortality, the presence of *Candida* in the lower respiratory tract may be of particular relevance in COVID-19 compared to other conditions requiring hospitalisation and admission to ICU. In line with this approach, other authors have reported information on rates of presence of *Candida* in the lower respiratory tract of patients with COVID-19 [23–25].

Regarding antibiotic resistance, the rates observed in this study in most of the bacterial pathogens, especially in *Enterobacterales* and *S. aureus,* were very high, greater than those observed in Italy in hospitalised patients with invasive infections prior to the COVID pandemic [26]. Although the high level of antibiotic resistance might be associated to the severity of the patients’ conditions and to the aggressive medical treatment, it must be considered that the adverse impact of the pandemic on the organization of healthcare systems may have interfered with the routine application of the practices of prevention and control of the transmission of MDRO (screening at admission, isolation of carriers etc.) [27]. Rates of antibiotic resistance were higher in Central/Southern Italy than in North Italy, mirroring a trend already described in the country [26].

Several recent studies examined the occurrence of co-infections or secondary infections in severely ill COVID-19 patients and their impact on mortality. While infections have a negative impact on COVID-19 patients’ survival in general [28], the acquisition of a nosocomial pathogen may have no or limited impact on mortality when only critically ill patients that require mechanical ventilation are considered [16, 23, 29]. In our study, however, clinicians reported either co-infections or secondary infections as a contributing cause of death in 36.3% of the death certificates.

The present results should be interpreted considering potential limitations. First, they are based on patients who died in hospital, without considering deaths occurring at home or in long-term care facilities and hospitalised patients who survived. We might have therefore selected a population with worse clinical conditions, more severe COVID-19 manifestations, and/or more aggressive infections. In addition, in performing a retrospective analysis, a selection bias might have occurred. Compared to the larger sample of patients with superinfections deceased in hospital with COVID-19 of the ISS mortality database (Additional File 1, Table S1), our sample had minor differences in terms of age, sex, and comorbidities, but a higher rate of admission to ICU (70.7% vs. 45.5%). This difference can be due to exclusion from our sample of less severe infections such as urinary tract or skin infections or those due to microorganisms commonly regarded as contaminants.

Since the study was retrospective, there was no harmonisation of diagnostic criteria for infections, sampling procedures or laboratory methods. In addition, the diagnosis of bacterial LRTI using established criteria [30] can be problematic, especially in intubated ICU patients with serious COVID-19 pneumonia, and it cannot be excluded that patients were colonized and not infected.

## Conclusions

Despite the above limitations, we believe that our data are informative in terms of type and frequency of co-infections and secondary infections occurring in hospitalised patients with COVID-19, and might be helpful in the design of preventive strategies against infections in Covid-19. The consequences of the COVID-19 emergency on the burden of hospital-acquired infections and MDRO are not immediate and will only become evident with time.

## Supplementary Information

**Additional file1.** **Table S1.** Characteristics of 1390 patients with superinfection from the ISS mortality database of patients deceased in hospital with COVID-19.

## Data Availability

The datasets generated and analysed during the current study are not publicly available due to confidentiality issues but are available from the corresponding author on reasonable request.
