# Supplementary material for: Microbiologically confirmed infections and antibiotic-resistance in a national surveillance study of hospitalised patients who died with COVID-19, Italy 2020–2021
Source: Antimicrob Resist Infect Control. 2022 May 21;11:74. doi: 10.1186/s13756-022-01113-y (PMC9123740; doi:10.1186/s13756-022-01113-y)
Supplement: Supplementary file 1 — Additional file1. Table S1. Characteristics of 1390 patients with superinfection from the ISS mortality database of patients deceased in hospital with COVID-19. [file 13756_2022_1113_MOESM1_ESM.docx]

**Additional file 1**

**Table S1 (supplementary materials). Characteristics of 1390 patients with superinfection deceased in hospital with COVID-19 of the ISS mortality database**

| **Demographics** |  |
| --- | --- |
| Age, median [interquartile range] | 77.0 [66-85] |
| Male gender (n, %) | 849 (61.1) |
| **Admission to Intensive Care Unit (n, %)** | 618 (45.5) |
| **Comorbidities (n, %)** |  |
| Chronic respiratory disease | 296 (21.5) |
| Active cancer in the past 5 years | 247 (18.0) |
| Diabetes | 413 (30.0) |
| Chronic renal failure | 317 (23.0) |
| **Patient location before admission (n, %)** |  |
| Home | 336 (56.9) |
| Other hospital | 142 (24.0) |
| Long-term care facility | 91 (15.4) |
| Other | 20 (3.4) |
